# Hepatitis B virus X protein specially regulates the sialyl lewis a synthesis among glycosylation events for metastasis

**DOI:** 10.1186/1476-4598-13-222

**Published:** 2014-09-25

**Authors:** Tae-Wook Chung, Seok-Jo Kim, Hee-Jung Choi, Kwon-Ho Song, Un-Ho Jin, Dae-Yeul Yu, Je-Kyung Seong, Jong-Guk Kim, Keuk-Jun Kim, Jeong-Heon Ko, Ki-Tae Ha, Young-Choon Lee, Cheorl-Ho Kim

**Affiliations:** Molecular and Cellular Glycobiology Laboratory, Department of Biological Science, SungKyunKwan University, 300 Chunchun-Dong, Jangan-Gu, Suwon, Kyunggi-Do 440-746 South Korea; Aging Research Center, Korea Research Institute of Bioscience and Biotechnology, Daejeon, 305-806 South Korea; Department of Veterinary Anatomy and Cell Biology, College of Veterinary Medicine and Agricultural Biotechnology, Seoul National University, Seoul, 151-742 South Korea; Department of Microbiology, Kyungpook National University, Daegu, 702-701 South Korea; Department of Clinical Pathology, TaeKyeung University, Gyeongsan, 712-719 South Korea; Systemic Proteomics Research Center, Korea Research Institute of Bioscience and Biotechnology, Yusong-Gu, Taejon, 305-600 South Korea; Division of Applied Medicine, School of Korean Medicine, Pusan National University, Yangsan, Gyeongsangnam-Do 626-770 South Korea; Faculty of Biotechnology, Dong-A University, Saha-Gu, Busan, 604-714 South Korea

**Keywords:** Hepatocellular carcinoma, Hepatitis B virus, Sialyl lewis antigen, E-selectin, Endothelial cells

## Abstract

**Background:**

The metastasis of hematogenous cancer cells is associated with abnormal glycosylation such as sialyl lewis antigens. Although the hepatitis B virus X protein (HBx) plays important role in liver disease, the precise function of HBx on aberrant glycosylation for metastasis remains unclear.

**Methods:**

The human hepatocellular carcinoma tissues, HBx transgenic mice and HBx-transfected cells were used to check the correlation of expressions between HBx and Sialyl lewis antigen for cancer metastasis. To investigate whether expression levels of glycosyltransferases induced in HBx-transfected cells are specifically associated with sialyl lewis A (SLA) synthesis, which enhances metastasis by interaction of liver cancer cells with endothelial cells, ShRNA and siRNAs targeting specific glycosyltransferases were used.

**Results:**

HBx expression in liver cancer region of HCC is associated with the specific synthesis of SLA. Furthermore, the SLA was specifically induced both in liver tissues from HBx-transgenic mice and in in vitro HBx-transfected cells. HBx increased transcription levels and activities of α2-3 sialyltransferases (ST3Gal III), α1-3/4 fucosyltransferases III and VII (FUT III and VII) genes, which were specific for SLA synthesis, allowing dramatic cell-cell adhesion for metastatic potential. Interestingly, HBx specifically induced expression of N-acetylglucosamine-β1-3 galactosyltransferase V (β1-3GalT 5) gene associated with the initial synthesis of sialyl lewis A, but not β1-4GalT I. The β1-3GalT 5 shRNA suppressed SLA expression by HBx, blocking the adhesion of HBx-transfected cells to the endothelial cells. Moreover, β1-3GalT 5 silencing suppressed lung metastasis of HBx-transfected cells in in vivo lung metastasis system.

**Conclusion:**

HBx targets the specific glycosyltransferases for the SLA synthesis and this process regulates hematogenous cancer cell adhesion to endothelial cells for cancer metastasis.

**Electronic supplementary material:**

The online version of this article (doi:10.1186/1476-4598-13-222) contains supplementary material, which is available to authorized users.

## Background

Metastasis consists of a series of sequential steps, such as shedding of cells from a primary tumor into circulation, survival of the cells in circulation, arrest in a new organ, extravasation into the surrounding tissue, initiation and maintenance of growth, and vascularization of the metastatic tumor [1]. Furthermore, the adhesion of malignant cells to the vascular endothelium is an important step for extravasation into target tissues of a new organ [2]. Malignant transformation is associated with abnormal glycosylation, resulting in the synthesis and expression of altered carbohydrate determinants on protein or lipid [3]. Expression of carbohydrate determinants, such as SLA and sialyl lewis X (SLX), is markedly enhanced in cancer cells [4, 5]. Also, SLA and SLX are involved in E-selectin-mediated adhesion of cancer cells to vascular endothelium, and these determinants play important roles in the hematogenous metastasis of cancer cells [4, 6]. HBV induces acute and chronic hepatitis, and is closely associated with the incidence of human liver cancer. Among the four proteins that originate from the HBV genome, HBx has been reported to be associated with hepatocellular carcinogenesis [7, 8]. Moreover, HBx induces liver cancer in transgenic mice [9, 10]. Recently, we have found that HBx causes the progression of liver cancer through the reduced expression of tumor suppressor PTEN [11]. Furthermore, we have reported that the enhanced expression of MMP-9 is eventually associated with the invasive potential of liver cells [12]. Previous studies show that HBx is potentially associated with hepatocellular carcinogenesis and invasion [11–14]. However, the precise function of HBx in the metastasis of liver cancer remains unclear at this time. Moreover, although HBV induces aberrant glycosylation-coupled hepatocellular carcinogenesis [15], some questions remain as to how the HBV functions on the aberrant glycosylation and selectively occupies its specific glycosylation targets for carcinogenesis and malignant metastasis.

In this study, we identified a relationship between HBx and SLX/A in liver cancer patients, and investigated whether HBx induces the expression of SLX/A in liver cells and HBx-transgenic mice. SLA-synthetic glycosyltransferase genes were transcriptionally up-regulated by HBx transfection in human normal Chang cells in vitro. Additionally, RNA-interference mediated depletion of SLA-synthesis machinery reduced HBx-dependent adhesion of liver cancer cells to endothelial cells and tumor metastasis in vivo. Together, it was demonstrated that HBx for metastatic potential is to select the specific expression of A type among sialyl lewis antigens, and finally, for interaction with E-selectin on the endothelial cell surface.

## Materials and methods

### Specimens

Human hepatocellular carcinomas (*n* = 11) used in this study were obtained from the department of Pathology, Yeungnam University Hospital. The protocols outlined in the following text were approved by the Ethics Committee of Yeungnam University Hospital.

### Animals

Paraffin blocks of liver tissues from transgenic mice carrying the HBx gene at the age of 3, 9 and 13 months used this study were obtained from Dr. Yu [10].

### Cell culture and transfection

Chang cells (ATCC number CCL-13), a human liver cell line and HBx-transfected cells were maintained using Dulbecco’s modified Eagle’s medium (DMEM) supplemented with 10% fetal bovine serum (FBS) at 37°C in a humidified 5% CO_2_ incubator. To investigate the relationship between HBx and carbohydrate ligand expression, the 465-bp cDNA encoding the open reading frame for HBx was inserted into the pcDNA3 expression vector at the *Hin*dIII/*Kpn*I sites. The cells were used for stable transfection with HBx using LipofectAMINE (Invitrogen) reagent following the manufacturer’s instructions. Transfected cells were then selected using cell culture medium containing 500 μg/ml G418 sulfate. Human umbilical vein endothelial cells (HUVECs) were obtained from Cambrex Bio Science (MD, USA), and were cultured in sterile endothelial growth medium (EGM-2, Cambrex Bio Science) and were maintained at 37°C in a humidified 5% CO_2_ incubator. Passages 5 to 8 of HUVECs were used in monolayer cell adhesion assay.

### Reverse transcription-polymerase chain reaction (RT-PCR)

Total RNA from each cell was isolated using the Coresol reagent (Corebio. Co., Seoul, Korea), and the cDNAs were synthesized by reverse transcriptase with an oligo dT-adaptor primer from a RNA PCR kit (Bioneer, Daejon, Korea) according to the manufacturer’s recommended protocol. The cDNA was amplified by PCR with primers, as shown in Additional file 1: Table S1. The use of equal amounts of mRNA in the RT-PCR assays was confirmed by analyzing the expression levels of β-actin. The PCR products were separated by gel electrophoresis on 2% agarose containing ethidium bromide with 1× TAE buffer.

### Immunohistochemistry

The liver tissues of non-transgenic mice (C57BL/6) and HBx-transgenic mice, normal liver tissues and liver cancer tissues of HBV-non infected and HBV-infected patients were fixed in 3.7% formalin and embedded in paraffin, and were then cut into 4 μm serial sections. The sections and HBx-transfected cells were immunostained with HBx, SLX and SLA antibodies, visualized with Dako EnVision kit (Dako, USA), and counterstained with hematoxylin.

### Monolayer cell adhesion assay

HUVECs were confluently cultured in 12 well plate and stimulated with or without TNF-α (10 ng/ml) for 8 h. Chang, Chang-pcDNA and Chang-pcDNA-HBx cells (5 × 10^5^cells/well) stained with DAPI were added on a monolayer culture of TNF-α-unstimulated or stimulated HUVECs, incubated for 20 min at 37°C with rotation at 60 rpm in shaking incubator, and washed to exclude non-specific cell to cell interaction. The attached cells were visualized using a microscope. To check the antibody-mediated inhibition of cell adhesion, the TNF-α-stimulated HUVECs were incubated with E-selectin (BD Biosciences) antibodies for 4 h at 37°C in humidified CO_2_ incubator, and each cell was then added to monolayer-cultured TNF-α-stimulated HUVECs. To block cell adhesion by SLA or SLX antibody, each cell was incubated with the antibody for 1 h and was then added to the TNF-α-induced HUVECs.

### Analysis of surface SLX and SLA by flow cytometry

The surface expression of SLX and SLA on each cell type was analyzed by FACS Calibur (Becton Dickinson, USA) using mouse anti- SLX and SLA after 24 h culture. The expression of SLX and SLA was analyzed by staining cells with monoclonal anti- SLX and SLA antibodies and then with goat anti-mouse IgM and IgG-FITC. The levels of antigen synthesized on the cell surface were expressed as the mean fluorescence intensity (MFI). Each experiment was repeated several times and the values represent a mean of two independent determinations.

### Preparation and transfection of small interfering RNA (siRNA) and small hairpin RNA (shRNA)

Each siRNA duplex was designed to target the coding sequence of human ST3Gal III, FUT III and VII mRNA, and plant chlorophyll a/b-binding protein mRNA was used as a negative control, and synthesized by Bioneer corp. (Daejeon, Korea). The target sequences of ST3Gal III, FUT III and VII siRNAs are 5′-CCUGCUGAAUUAGCCACCAdTdT-3′, 5′-CACACUCAGGUGACCUACAdTdT-3′ and 5′-CCCUGAACAAAUCUUGGGUdTdT-3′ respectively. HBx-transfected cells were co-transfected with ST3Gal III, FUT III, VII siRNAs and negative control siRNA respectively by using LipofectAMINE (Invitrogen) according to the manufacturer’s instructions. One day after transfection, the cells were used for experiments. In order to silence with β-1, 3 GalT 5 gene, three pairs of oligonucleotides were designed by the siRNA target finder (Ambion). Different regions of human β-1,3 GalT 5 gene were annealed and cloned in the pSilencer™ 3.1-H1 puro expression vector (Ambion, Austin, TX) digested with *Hind*III/*BamH*I double digested pMEHMpuro. Three target sequences were; (1) AAGGGAAAGCAGCTGAAGACA, (2) AAGTGGTTTGTCAGTAAATCT, (3) CTCGAAAGGCTGAACATCAGATTGG. After transfection with shRNA vectors using the Welfect method (Welgene, Daegu, Korea), cells were selected by puromycin (250 ng/mL) and used in the experiments.

### Reporter plasmids and luciferase assay

For preparation of human ST3Gal III, FUT III, VII and β-1,3 GalT 5 promoters, transcriptional start points of each gene were identified and the 5′-flanking sequence was obtained. PCR amplification from the genomic DNA of HBx-transfected Chang cells was performed with EF-*Taq* polymerase (SolGent, Seoul, Korea) using primers, as shown in Additional file 1: Table S2. The amplified fragments of each gene were sequenced using pT7Blue(R) T-vector (Novagen) and were inserted into the pGL3-Basic vector (Promega, Madison, WI) digested with each restriction enzyme. After co-transfection with each-luciferase reporter plasmid and β-galactosidase reporter plasmid, cells were rinsed in PBS, and lysed in Luciferase Lysis Buffer (Promega). Luciferase activities were measured using the Luciferase assay system (Promega) and Luminoskan Ascent (Thermo Labsystems, Helsinki, Finland). Luciferase activity was normalized with the β-galactosidase activity in each cell lysate. Data were represented as the mean from three independent experiments.

### Immunofluorescence microscopy

To confirm the enhanced expression of carbohydrate ligands on the liver cell surface by HBx, Chang cells and HBx-transfected cells were seeded at a sub-confluent density on sterile coverslips in 6-well tissue culture plates. After incubating the attached cells in DMEM containing 10% FBS for 24 h, they were fixed in 3.7% formalin and washed 3 times with PBS. Non-specific sites were then blocked with 5% bovine serum albumin-containing PBS for 30 min at room temperature with gentle rocking. Thereafter, a solution of SLX and SLA antibodies was flooded over the cells and the cultures were incubated at 4°C overnight. After washing with PBS, the cells were further incubated with FITC-conjugated goat anti-mouse IgM and IgG for 1 h at room temperature, followed by washing with PBS, and were then analyzed using fluorescence microscopy. The pre-absorbed primary antibody or the secondary antibody alone was also applied as a negative control experiment.

### Lung metastasis assay

Chang-HBx cells, pSilencer vector-transfected Chang-HBx cells and β-1,3Gal T5 shRNA-tranfected Chang-HBx cells (5 × 10^5^) in 10 μl PBS were injected into the tail vein of 8-week-old female BALB/c nude mice (*n* = 6-8). The mice were cared for in accordance with the national and internationals rules of Korea for animal studies. 35 days after injection with cells, the mice were euthanized, and lungs from each mice were isolated. The isolated lung tissues were fixed in 10% formalin and embedded in paraffin, and were then stained with hematoxylin and eosin prior to determination.

## Results

### The relationship between HBx and SLA in HCC patients

**Figure 1:**
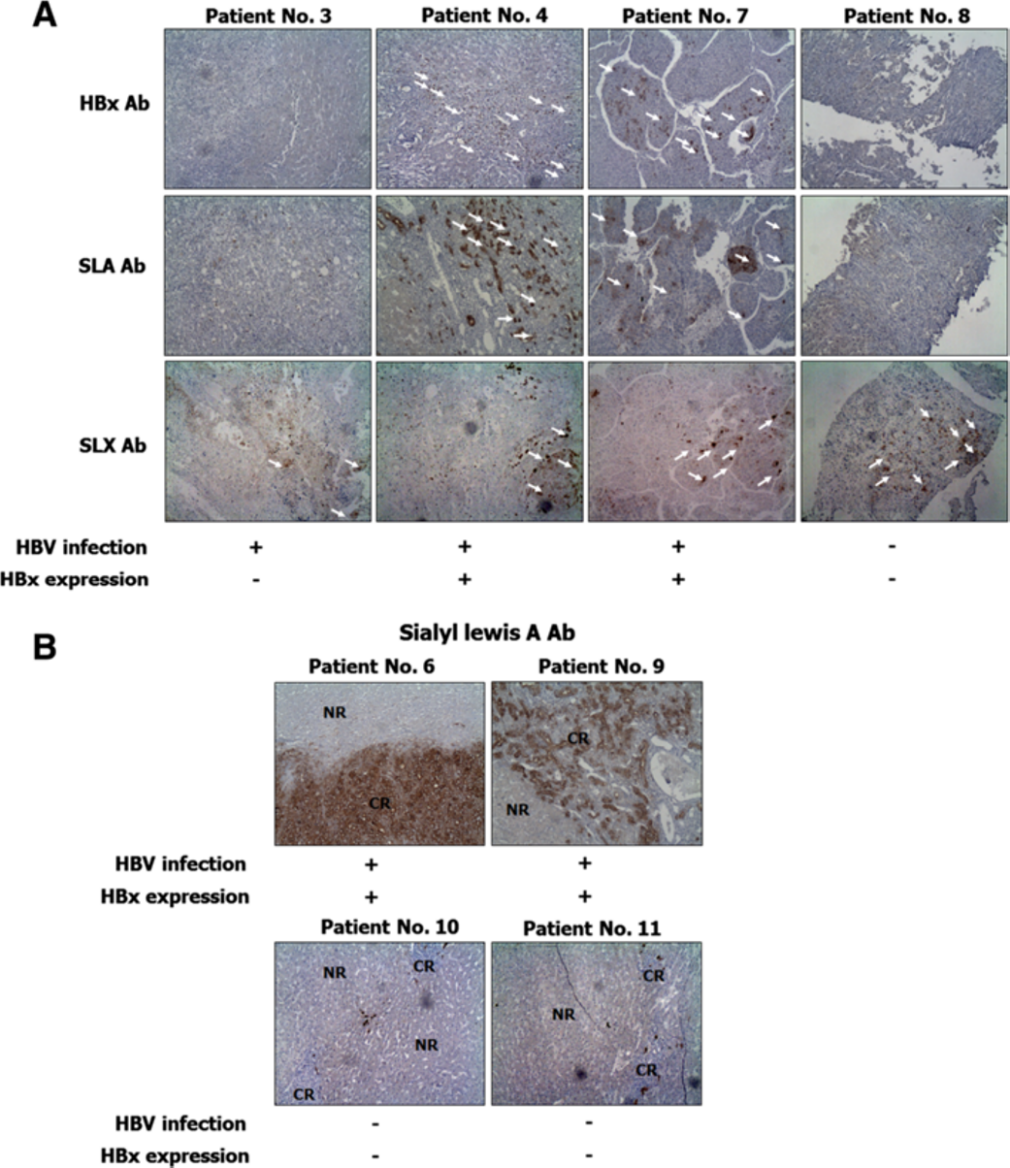
**The expression of SLA in cancer region of HBx-expressed HCC patients.** Normal liver tissues and liver cancer tissues of HBV-non infected and HBV-infected patients were fixed in 3.7% formalin and embedded in paraffin, and then they were cut into 4 μm serial sections. The sections were immunostained with HBx, SLX and SLA antibodies, visualized with Dako EnVision kit (Dako, USA), and counterstained with hematoxylin. **(A)** Dark brown staining indicates the expression of HBx, SLX and SLA in the cancer region of HBV-non infected or HBV-infected patients. **(B)** SLA expression in cancer region (CR) and normal region (NR) of HCC patients is stained as dark brown.

An increased expression of SLX and SLA structures in various malignancies and in metastatic lesions has been well documented [4, 5, 16, 17]. To determine whether HBx expression in liver cancer is associated with SLX/A expression, we performed immunohistochemistry using liver tissues obtained from 11 HCC patients (10 males and 1 female) between the ages of 44 and 63. As shown in Table 1 and Figure 1A, although SLX was highly expressed in liver cancer tissues, HBx expression in HBV-infected HCC was not associated with its expression. However, as shown in Table 1 and Figure 1A and B, HBx expression in the cancer region of HBV-infected HCC was more related to SLA expression than that of HBV-uninfected HCC and HBx no-expression in HBV-infected HCC, except in the case of patient No. 2. Moreover, as shown in Figure 1B, SLA expression was increased in the cancer region of HCC compared to the normal region. These results suggest that HBx might induce the formation of SLA in the cancer region.

**Table 1:** **The relationship between HBx and SLX/A in HCC patients**

Patient No.	Sex	Virus	HBs antigen	HBx expression	Outcome	SLX	SLA
	(Age)					SLX expression	SLA expression
1	M (63)	-	-	-	HCC	+	+
2	M (57)	HBV	+	-	HCC	+++	++
3	M (55)	HBV	+	-	HCC	++	+
4	M (44)	HBV	+	+	HCC	++	+++
5	M (55)	HBV	+	+	HCC	+++	++
6	M (62)	HBV	+	+	HCC	+++	+++
7	F (56)	HBV	+	+	HCC	++	++
8	M (59)	-	-	-	Cirrhosis/HCC	+	-
9	M (46)	HBV	+	+	HCC	++	+++
10	M (62)	-	-	-	HCC	++	+
11	M (49)	-	-	-	HCC	++	+

Results of SLX and SLA expression detected by immunohistochemistry are given according to the distribution of positive hepatocytes in cancer region: -, no staining; +, weak staining (<5% hepatocytes); ++, strong staining (>5% ~ <20% hepatocytes); +++, very strong staining (>20% hepatocytes). M, male; F, female; -, no detection; ×, no expression; O, expression.

### The enhanced expression of SLA in HBx-transgenic mice

**Figure 2:**
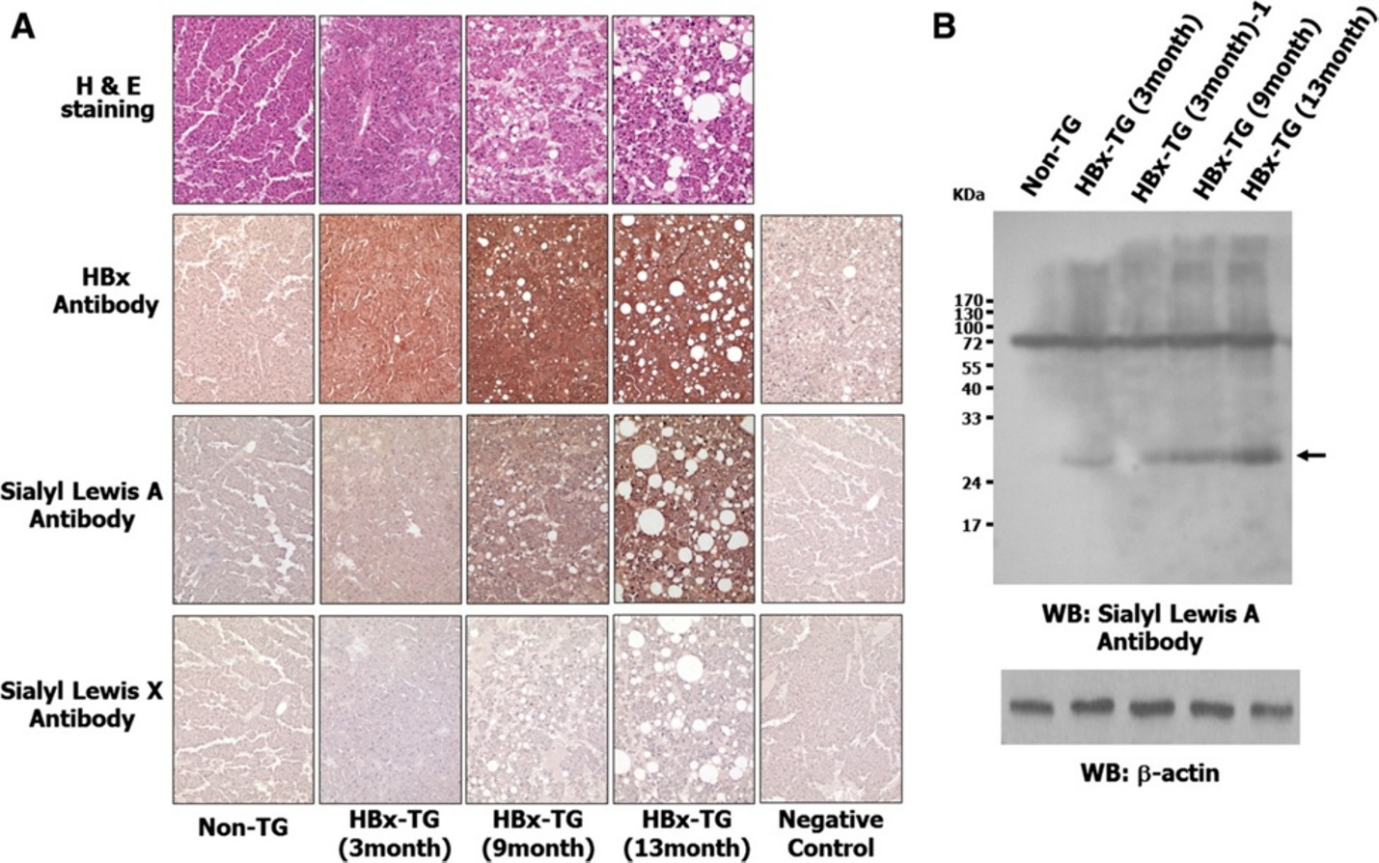
**The expression of SLA antigen in HBx-transgenic mice. (A)** The liver tissues of non-transgenic mice (C57BL/6) and HBx-transgenic mice were fixed inn 3.7% formalin and embedded in paraffin, and were then cut into 4 μm serial sections. The serial sections were immunostained with HBx, SLX and SLA antibodies, visualized with Dako EnVision kit (Dako, USA), and counterstained with hematoxylin. Negative controls were immunostained without the primary antibody. **(B)** Total proteins (30 μg) from each liver tissue were isolated and immunoblotting analysis was performed with the indicated antibodies.

Previously, Yu group has reported that HCC frequently occurred in HBx-transgenic mice [9]. Furthermore, several researchers have reported HBx function in hepatocarcinogenesis using these mice. Thus, we examined whether HBx is associated with the expression of sialyl lewis antigens in HBx-transgenic mice. The dysplastic liver region of HBx transgenic mice at the age of 13 months showed small neoplastic nodules and grossly identified HCCs, which was consistent with the results of a previous report [10]. As shown in Figure 2, in liver tissue from 9 and 13 month old HBx-transgenic mice, H&E staining showed mild to severe hepatic necrosis, fatty change, mild to moderate chronic hepatitis and cytoplasmic vacuolation, compared to normal mice and 3 month old HBx-transgenic mice. Furthermore, liver tissue from 13 month old HBx-transgenic mice showed that the nuclei/cytoplasm ratio was increased. Immunostaining against HBx antibody showed that HBx was only expressed in HBx-transgenic mice (Figure 2). Interestingly, SLA expression was highly detected in liver tissue from 13 month old HBx-transgenic mice. However, SLX was rarely expressed in liver tissue from normal or HBx-transgenic mice. These results suggest that HBx is associated with SLA expression in liver cancer.

### Enhanced expressions and transcriptional activities of α2-3 sialyltransferases and α1-3/4 fucosyltransferases by HBx

Fucosyltransferases and α2-3 sialyltransferases are required for SLX and SLA synthesis (Additional file 1: Figure S1) [3]. Thus, we checked the expression patterns of α2-3 sialyltransferases (ST3Gal I-VI) and α1-3/4 fucosyltransferases (FUT III-VII) in HBx-transfected cells. As shown in Additional file 1: Figure S2A, HBx obviously up-regulated mRNA expression of ST3Gal III, one of six α2-3 sialyltransferase isoenzymes. Among five fucosyltransferases (FUT III-VII), HBx markedly increase mRNA levels of FUT III and VII genes (Additional file 1: Figure S2A). As HBx regulates mRNA levels of ST3Gal III, FUT III and VII genes, the transcription activities of these genes were assessed using the luciferase reporter assay system in pcDNA and HBx-transfected Chang cells. As shown in Additional file 1: Figure S1B, the transcriptional activities of the ST3Gal III, FUT III and VII promoters were significantly increased in HBx-transfected Chang cells, as compared with the control (pcDNA-transfected Chang cells). These results suggest that HBx modulates transcription levels of ST3Gal III, FUT III and VII genes for SLX and SLA synthesis.

### The induction of SLA in HBx-transfected cells and HBx-transfected cell adhesion to endothelial cells

**Figure 3:**
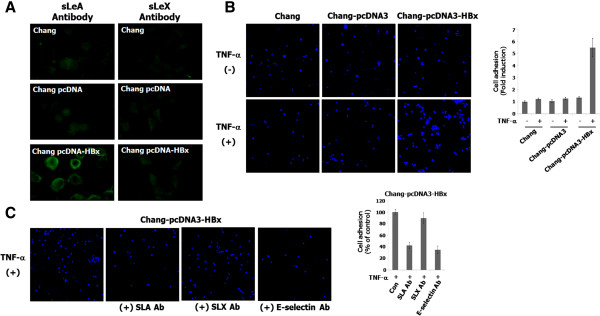
**The increase of SLA determinant in HBx-transfected cells and the adhesion of HBx-transfected cells to endothelial cells. (A)** The cells were fixed, and then immunostained with SLA and SLX antibodies. The immunostained cells were analyzed using a fluorescence microscope. **(B)** Chang, Chang-pcDNA and Chang-pcDNA-HBx cells (5 × 10^5^ cells/well) were added on TNF-α-unstimulated or stimulated HUVECs, and were incubated for 20 min at 37°C with rotation at 60 rpm in a shaking incubator. The attached cells were visualized using a microscope. **(C)** To check the antibody-mediated inhibition of cell adhesion, the TNF-α-stimulated HUVECs were incubated with E-selectin antibody for 4 h at 37°C in a humidified CO_2_ incubator, and each cell was then added to monolayer-cultured TNF-α-stimulated HUVECs. To block cell adhesion by SLA or SLX-specific antibody, each of cells was incubated with the antibody for 1 h and added to the TNF-α-induced HUVECs.

SLX and SLA determinants are synthesized by α2-3 sialyltransferases, including ST3Gal I-VI and α1-3/4 fucosyltransferases, such as FUT III-VII, in several types of cancer cells [18–23]. We further investigated whether HBx results in the stimulation of SLX and SLA expression in liver cells. As shown in Figure 3A, immunostaining against SLA clearly showed an increase in HBx-transfected Chang cells compared to Chang and Chang pcDNA cells, but not SLX. This result indicates that HBx induces SLA expression. SLA or SLX determinant synthesized on surface of cancer cells and E-selectin expressed on endothelial cells induced by inflammatory stimulus such as TNF-α, IL-1α or IL-1β are required for hematogenous metastasis because the adhesion of malignant cells to the vascular endothelium is an important step for extravasation into target tissues of a new organ [4–6]. Thus, we investigated whether HBx-transfected cells are attached to TNF-α-stimulated endothelial cells. As shown in Figure 3B, the adhesion of Chang-pcDNA-HBx cells to TNF-α-treated endothelial cells was clearly increased compared to the adhesion of Chang, Chang-pcDNA and Chang-pcDNA-HBx cells to TNF-α-untreated endothelial cells, as well as Chang and Chang-pcDNA cells to TNF-α-activated endothelial cells. We further checked whether the SLA expression on HBx-transfected cells and E-selectin expressed on TNF-α-stimulated endothelial cells is closely associated with cancer cell to endothelial cell interactions for metastatic potential, using antibodies against SLX/A and E-selectin. As shown in Figure 3C, the enhanced adhesion of Chang-pcDNA-HBx cells to TNF-α-treated endothelial cells is markedly inhibited by treatment with anti-SLA and E-selectin antibodies, respectively, but not by treatment with anti-SLX antibody. These results clearly indicate that SLA expressed by HBx on liver cancer cells is critical for cell-to-cell adhesion by binding it to E-selectin expressed on TNF-α-stimulated endothelial cells.

### The inhibition of SLA expression in HBx-transfected cells and HBx-tranfected cell adhesion to endothelial cells by ST3Gal III, FUT III and FUT VII siRNAs

**Figure 4:**
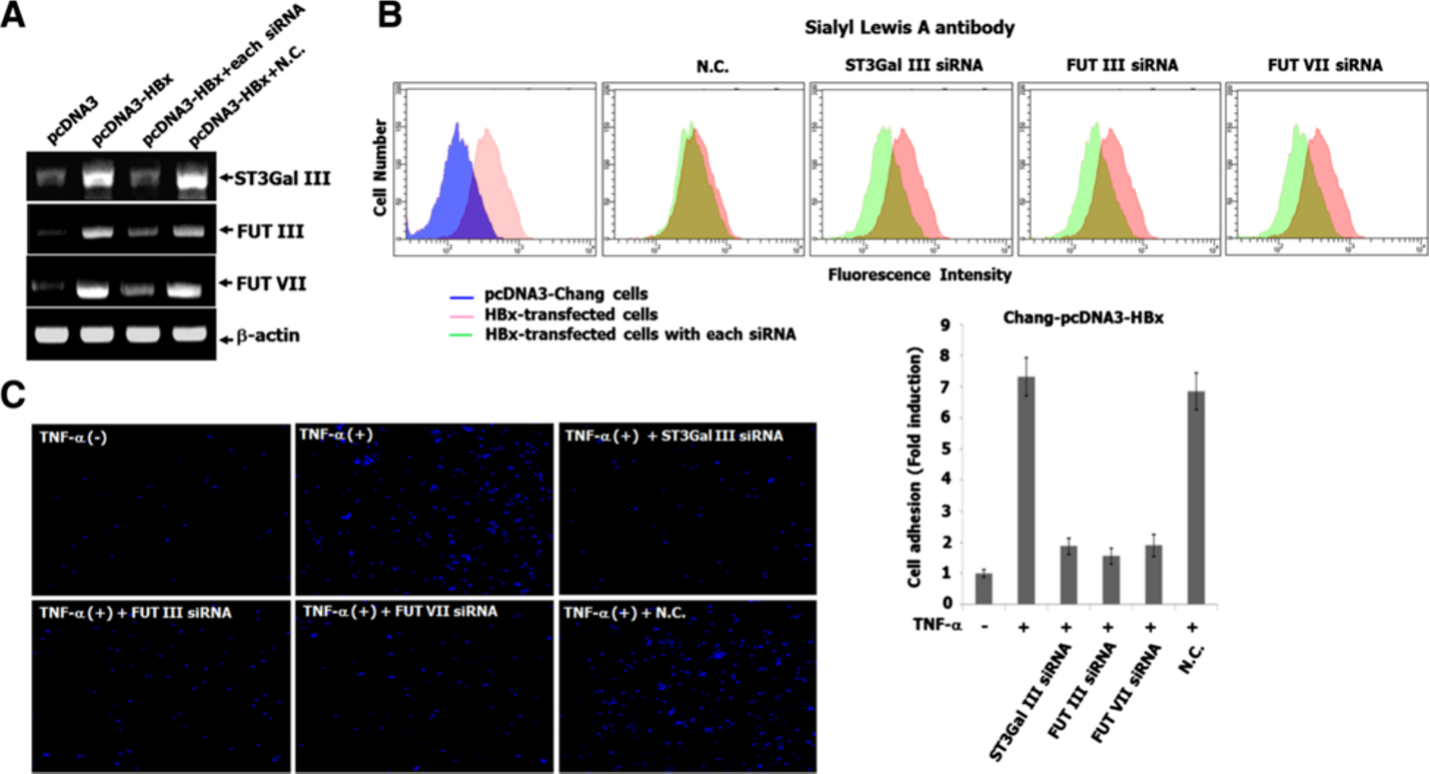
**The inhibition of SLA expression in HBx-transfected cells and HBx-tranfected cell adhesion to endothelial cells by ST3Gal III, FUT III and FUT VII siRNAs. (A)** Each cell was co-transfected with ST3Gal III, FUT III and VII siRNAs, as well as negative control siRNA, respectively. Total RNA from these cells was isolated. The mRNA expression of ST3Gal III, FUT III and VII were detected by RT-PCR, using primers which are indicated in Table 1. β-actin was included as an internal control. **(B)** To check whether SLA expression induced in HBx-transfected cells was suppressed by the siRNAs against ST3Gal III, FUT III and FUT VII genes, the expression was examined using flow cytometric analysis and immunofluorescent staining. Fluorescence histograms of a representative experiment indicate the baseline expression of SLA. The y-axis gives the percentage of positive cells (counts); the x-axis gives fluorescence intensity. **(C)** Each cell was co-transfected with ST3Gal III, FUT III and VII siRNAs and negative control siRNA (N.C.), respectively. Each cell was added on TNF-α-stimulated HUVECs, and was incubated for 20 min at 37°C with rotation at 60 rpm in a shaking incubator. The attached cells were visualized using a microscope, as described in Materials and Methods.

To clarify whether induction of ST3 Gal III, FUT III and FUT VII expression by HBx is essential for SLA expression in liver cancer cells, the expression of these enzymes was depleted using RNA-interference strategy in HBx-expressing cells. As shown in Figure 4A, the increased transcription of ST3Gal III, FUT III and FUT VII genes in HBx-tansfected cells was inhibited by the transient transfection of ST3Gal III, FUT III and FUT VII siRNAs, respectively. Furthermore, as shown in Figure 4B, silalyl lewis A expression induced in HBx-transfected cells was diminished by the siRNAs against ST3Gal III, FUT III and FUT VII genes, respectively, as evidenced by immunofluorescence staining and flow cytometric analysis. These results suggest that the induction of ST3Gal III, FUT III and FUT VII expression by HBx is essential for the production of SLA determinant in liver cancer.

We further investigated whether the enhanced adhesion of Chang-pcDNA-HBx cells to TNF-α-stimulated endothelial cells is obstructed using siRNAs against ST3Gal III, FUT III and FUT VII genes. As shown in Figure 4C, the increased adhesion of Chang-pcDNA-HBx cells to TNF-α-stimulated endothelial cells was clearly suppressed by the siRNAs against ST3Gal III, FUT III and FUT VII genes, but not negative siRNA. These results indicate that SLA determinant, synthesized through the induced expression of ST3Gal III, FUT III and FUT VII by HBx in liver cells, binds to E-selectin induced by inflammatory cytokines in endothelial cells.

### The suppression of cell adhesion to endothelial cells and lung metastasis in HBx-transfected cells with β1,3-galactosyltransferase shRNAs

**Figure 5:**
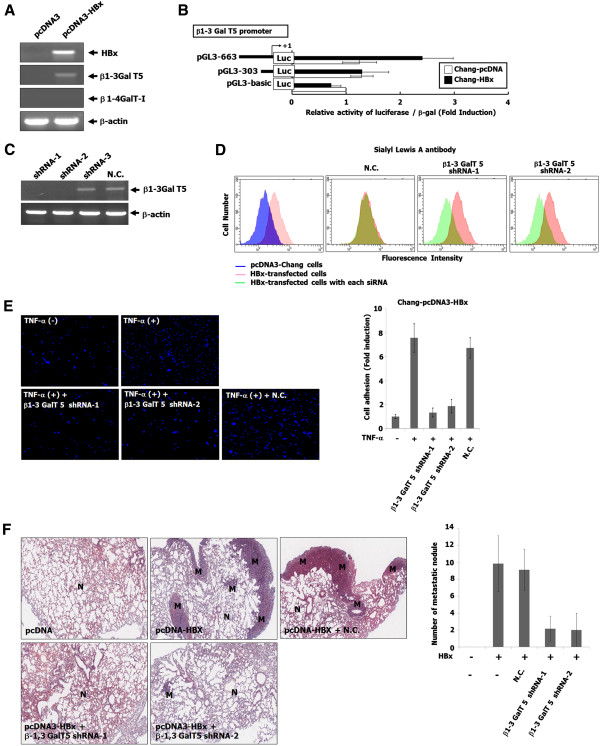
**The inhibition of cell adhesion to endothelial cells and metastasis through the suppression of SLA synthesis in HBx-transfected cells by β1,3-galactosyltransferase shRNA. (A)** Total RNA from each cell was isolated using the Trizol reagent. The mRNA expressions of β1-3 and 4 galactosyltransferase genes were detected by RT-PCR using the primers indicated in Table 1. β-Actin was included as an internal control. **(B)** After transfection with β1-3Gal-T 5 promoter, luciferase activity from the cells was analyzed as described in Materials and Methods. Relative luciferase activity was normalized with the activity of pCMVβ-gal plasmid. **(C)** Each cell was transfected with the shRNA-1 and 2 against β1-3Gal-T 5 gene. Total RNA from these cells was isolated. The mRNA expression of β1-3Gal-T 5 gene was detected by RT-PCR using the primers indicated in Table 1. β-Actin was included as an internal control. **(D)** To check whether silalyl lewis A expression induced in HBx-transfected cells was suppressed by the shRNAs against β1-3Gal-T 5 gene, we examined this using flow cytometric analysis. Fluorescence histograms of a representative experiment indicate the baseline expression of SLA. The y-axis gives the percentage of positive cells (counts); the x-axis gives fluorescence intensity. **(E)** Each cell was transfected with the shRNA against β1-3Gal-T 5 gene. Each cell was added on TNF-α-stimulated HUVECs, and was incubated for 20 min at 37°C with rotation at 60 rpm in a shaking incubator. The attached cells were then visualized using a microscope. **(F)** Hematoxylin and eosin staining of the lungs. The stained sections of the lungs were photographed at 40× magnification. M, metastatic nodule; N, normal.

As shown in Additional file 1: Figure S1, at the initial step of the biosynthesis of SLA antigen, *N*-acetylglucosamine-β1-3 galactosyltransferase V (β1-3Gal-T 5) transfers galactose (Gal) to N-acetylglucosamine (GlcNac) with 1-3-linkage. This restricted distribution of Lewis type 1 antigens has been considered to result from the limited expression of β1-3Gal-T 5, which synthesizes the type 1 structure [24]. On the other hand, for the generation of SLX antigen, N-acetylglucosamine-β1-4 galactosyltransferase I (β1-4Gal-T I) catalyzes the addition of UDP-galactose to terminal N-acetylglucosamine with 1-4-linkage for the type 2 structure. [25]. Thus, we examined whether *N*-acetylglucosamine-β1-3/4 galactosyltransferases is regulated by HBx. Interestingly, HBx clearly induced the expression of β1-3Gal-T 5, but not β1-4Gal-T I (Figure 5A). Next, the transcription activity of β1-3Gal-T 5 was determined using a luciferase reporter assay system in pcDNA and HBx-transfected Chang cells. As shown in Figure 5B, the activity of the β1-3Gal-T 5 promoter was significantly increased in HBx-transfected cells compared to pcDNA-transfected cells. We further investigated whether SLA expression in HBx-transfected cells and the enhanced adhesion of Chang-pcDNA-HBx cells to TNF-α-stimulated endothelial cells are inhibited using shRNA against the β1-3Gal-T 5 gene. As shown in Figures 5C and D, the shRNA-1 and 2 against β1-3Gal-T 5 gene clearly suppressed β1-3Gal-T 5 mRNA and SLA expression in HBx-transfected cells. Furthermore, the increased adhesion of Chang-pcDNA-HBx cells to TNF-α-stimulated endothelial cells was clearly inhibited by the shRNA against β1-3Gal-T 5 gene (Figure 5E). Based on the suppressed adhesion of HBx-transfected cells to endothelial cells by β1-3Gal-T 5 shRNA, we further investigated whether the silencing of β1-3Gal-T 5 expression suppresses lung metastasis of HBx-transfected cells. As shown in the histologic results, micrometastatic lesions of lung isolated from HBx-transfected cells carrying β1-3Gal-T 5 shRNA-1 and 2 were dramatically reduced compared to those of HBx-transfected cells with and without negative control shRNA (Figure 5F). These results strongly suggest that the induction of β1-3Gal-T 5 expression by HBx, the first step of the synthesis of SLA, is induced by HBx for liver cancer metastatic potential.

## Discussion

SLA has been shown to be expressed in cancer cells derived from the digestive organs, including colon, rectum, pancreas and biliary tract. Furthermore, SLX has been reported to be highly expressed in breast, ovarian and pulmonary cancer cells [3]. Moreover, in serum, the SLX levels in patients with hepatocellular carcinoma were significantly higher than those of normal controls as well as those with benign liver cirrhosis [26]. Several studies indicated that SLX expression might be associated with hepatocellular carcinoma [27–29]. It has been reported that glycan-related gene expression related to the synthesis of N-glycan and glycolipids, particularly the sialyl Lewis antigen was induced in human metastatic hepatocellular carcinoma cells [30]. The induction of these carbohydrate antigens in some cancers is considered as risk factor for hematogenous metastasis. In this study, our results also showed that SLX expression in cancer regions of HCC patients was higher compared to non-cancer regions, except in 3 cases, in which SLX was also highly expressed in non-cancer regions. However, HBV infection and HBx expression in HCC patients was not correlated with SLX expression in cancer regions of HCC patients. Whereas, HBx expression in cancer regions of HBV-infected HCC was associated with SLA expression compared to HBV-uninfected HCC and HBx no-expression in HBV-infected HCC. Thus, we further investigated whether HBx expression lead the increased expression of sialyl lewis carbohydrate antigens in the liver cells and transgenic mice. Our results found that HBx had an effect on the formation of SLA determinant using HBx-transfected cells and HBx-transgenic mice.

Sialylated carbohydrate antigens, such as SLX and SLA, are divided into type 1 chain (Galβ1-3GlcNAc-R) and type 2 chain (Galβ1-4GlcNAc-R). The sequential addition of galactose (Gal), sialic acid (Sia) and fucose (Fuc) to *N*-acetylglucosamine (GlcNac) by glycosyltransferases is required for the biosynthesis of SLX and SLA. First of all, *N*-acetylglucosamine-β1-3 or β1-4 galactosyltransferase transfers a galactose to an *N*-acetylglucosamine with 1–3 or 1-4-linkage, resulting in the synthesis of type 1 chain or type 2 chain, and then galactose-α2-3 sialyltransferase transfers a sialic acid to the Gal residue of the type 1 or 2 chain with an 2–3 linkage. Finally, α1-4/3 fucosyltransferase transfers a fucose to the GlcNAc residue of the sialylated-type 1 or 2 chain with an 1-4/3 linkage to complete the synthesis of Siaα2-3Galβ1-3/4[Fucα1-4/3]GlcNAc structure for SLA or X [4, 5, 24]. Previous studies have indicated that α2-3 sialyltransferases (ST3Gal I-VI) and α1-3/4 fucosyltransferases (FUT III-VII) were increased for the synthesis of SLX and SLA determinant in various tumors. Thus, we examined whether HBx modulates the transcription of α2-3 sialyltransferase, α1-3/4 fucosyltransferase genes and *N*-acetylglucosamine-β1-3/4 galactosyltransferases. Our results showed that the transcriptional expressions and activities of ST3Gal III, FUT III and FUT VII genes were markedly induced by HBx in Chang cells. Moreover, to clarify whether HBx is required for the synthesis of SLA in liver cancer cells through the induction of ST3Gal III, FUT III and FUT VII expression, the siRNAs against ST3Gal III, FUT III and FUT VII genes were used in HBx-transfecte cells. Our study indicated that silalyl lewis A expression induced in HBx-transfected cells was diminished by the siRNAs against ST3Gal III, FUT III and FUT VII genes, respectively. These results suggest that the HBx-induced sialyltransferase and fucosyltransferase expression lead to the synthesis of SLA determinant in HBx-tansfected cells. In addition to transcriptional studies, our previous papers have shown that HBx activated AKT signaling through the down-regulation of PTEN expression [11], and regulated the expression and transcriptional regulation of MMP-9 by the activation of AP-1 and NF-κB via ERKs and PI-3 K-AKT pathways [12]. Promoters of ST3Gal III, FUT III, FUT VII and β-1,3 GalT5 related to synthesis of sialyl lewis A also contain AP-1 and NF-κB transcriptional factors as confirmed by Transcription Element Search System to predict the possible transcription factor binding sites in each promoter region (data not shown). However, the precise transcriptional regulations of these genes by HBx have not been characterized in the present study. These issues will be focused on our further study.

It has been well known that interactions between endothelial selectins and cancer cells regulate metastasis and E-selctin contributes to the adhesion of cancer cells to endothelial cells by interacting with SLX and SLA for hematogeneous metastasis. Furthermore, elevated levels of serum E-selectin in patients bearing SLX- and SLA-positive tumors has been shown to predict a high risk for developing metastasis. A previous report indicated that E-selectin expression is quite low in resting endothelial cells [3, 31]. However, E-selectin expression at the surface of endothelial cells is induced by tumor-derived IL-1α and monocyte-induced IL-1β in inflammatory metastatic sites [3, 32, 33]. Furthermore, inflammation can play a regulatory role in cancer progression and metastasis. Metastatic tumor cells entering the liver trigger a proinflammatory response involving Kupffer cell-mediated release of TNF-α and the up-regulation of E-selectin in vascular endothelial cells [34]. In this study, we confirmed the adhesion of HBx-transfected cells to TNF-α-stimulated vascular endothelial cells using a monolayer cell adhesion assay. To further clarify whether the interaction of SLA on HBx-transfected cells with E-selectin on TNF-α-induced endothelial cells is involved in HBx-transfected cell adhesion to endothelial cells stimulated by TNF-α, specific antibodies against SLX, SLA and E-selectin were used. Our data clearly indicated that treatment of HBx-transfected cells with SLA antibody, but not with SLX antibody, diminished cell to cell adhesion. Our previous data showed that HBx induced SLA expression in liver cells through the up-regulation of ST3Gal III, FUT III and FUT VII expression, Thus, we also checked whether the suppression of SLA synthesis in HBx-transfected cells by ST3Gal III, FUT III and FUT VII siRNAs, respectively, is associated with the inhibition of HBx-transfected cell adhesion to TNF-α-treated endothelial cells. The enhanced adhesion of HBx-transfected cells to TNF-α-stimulated endothelial cells was clearly decreased by the siRNAs against ST3Gal III, FUT III and FUT VII genes. These results indicate that SLA determinant synthesized by HBx-induced expression of ST3Gal III, FUT III and FUT VII in liver cells binds to E-selectin induced by TNF-α in endothelial cells, suggesting metastasis of HBV-infected primary liver cancer.

Our data clearly showed that the enhancement of SLA determinant in HBx-transfected cells was associated with the induction of ST3Gal III, FUT III and FUT VII expression. However, several reports demonstrate that the synthesis of SLX antigen is induced by the expression of ST3Gal III, FUT III and FUT VII genes in various tumors [3, 4]. We questioned why the expression of SLA but not SLX is induced by HBx in liver cells, and initially assumed that the expression of β1-3 galactosyltransferase, which transfers a galactose to an *N*-acetylglucosamine with 1-3-linkage for the synthesis of SLA antigen, is increased by HBx. Interestingly, our results showed that HBx markedly induced β1-3 galactosyltransferase expression but not that of β1-4 galactosyltransferase. The transcriptional activity of β1-3 galactosyltransferase mRNA was also increased in HBx-transfected cells. Furthermore, the β1-3 galactosyltransferase shRNA suppressed SLA antigen expressed by HBx in liver cells, and blocked the adhesion of HBx-transfected cells to TNF-α-stimulated endothelial cells. In the tumor metastasis assay, β1-3 galactosyltransferase shRNA significantly inhibited the metastasis of the tail vein-injected HBx-transfected cells to the lung in mice. Based on these findings, HBx increases SLA determinant by inducing β1-3 galactosyltransferase expression, resulting in liver cancer metastasis.

In conclusion, and as illustrated by Additional file 1: Figure S3, the crucial step for the extravasation of cancer metastasis is associated with the attachment of hematogenous cancer cells to the blood vessel endothelium. In this study, we demonstrate that HBV prefers the A type specific glycosyltransferases for the SLA synthesis in liver cancer, and that the synthesized SLA further interacts with E-selectin on endothelial cells. This process is the final destination of the hematogenous cancer cell-involved viral occupation to the host. Furthermore, we expect that the relationship between HBx expression and sialyl lewis A synthesis might be a clue of prognosis in malignancy of HBV-infected liver cancer patients. In addition, the inhibition of sialyl lewis A synthesis might be a therapeutic avenue for the enhanced HBx expression in HBV-infected liver cancer patients.

## Electronic supplementary material

Additional file 1: **Table S1.** The sequences of primers used in this study. **Table S2.** The sequences of primers for constructions of each promoter. **Figure S1.** The structure of SLA and SLX. For SLA synthesis, *N*-acetylglucosamine-β1-3 galactosyltransferase (β1-3Gal-T 5) transfers a galactose (Gal) to an N-acetylglucosamine (GlcNac) with 1-3-linkage, resulting in the synthesis of type 1 chain, and then galactose-α2-3 sialyltransferase transfers a sialic acid (Sia) to the Gal residue of the type 1 with an 2-3 linkage. Finally, α1-4/3 fucosyltransferase transfers a fucose (Fuc) to the GlcNAc residue of the sialylated-type 1 with an 1-4 linkage to complete the synthesis of Siaα2-3Galβ1-3[Fucα1-4]GlcNAc structure. However, if N-acetylglucosamine-β1-4 galactosyltransferase I (β1-4Gal-T I) catalyzes the addition of UDP-galactose to terminal N-acetylglucosamine with 1-4-linkage, resulting in the synthesis of type 2 chain, continuously, galactose-α2-3 sialyltransferase transfers a sialic acid to the Gal residue of the type 2 chain with an 2-3 linkage. Finally, α1-4/3 fucosyltransferase transfers a fucose to the GlcNAc residue of the sialylated-type 2 chain with an 1-3 linkage to complete the synthesis of Siaα2-3Galβ1-4[Fucα1-3]GlcNAc structure. **Figure S2.** The enhanced expression and promoter activity of ST3Gal III, FUT III and VII genes in HBx-transfected cells. (A) Total RNA from each cell was isolated using the Trizol reagent. The mRNA expression of α2-3 sialyltransferases and α1-3/4 fucosyltransferases genes was detected by RT-PCR using primers indicated in **Table 1**. β-actin was included as an internal control. 1, Chang; 2, Chang pcDNA; 3, Chang pcDNA-HBx. (B) After transfection with each promoter, luciferase activity from the cells was analysed as described in Materials and Methods. Relative luciferase activity was normalized with the activity of pCMVβ-gal plasmid. (DOC 568 KB)
