# Supplementary material for: Hepatitis B virus X protein specially regulates the sialyl lewis a synthesis among glycosylation events for metastasis
Source: Mol Cancer. 2014 Sep 25;13:222. doi: 10.1186/1476-4598-13-222 (PMC4190352; doi:10.1186/1476-4598-13-222)
Supplement: Supplementary file 1 — Additional file 1: Table S1. The sequences of primers used in this study. Table S2. The sequences of primers for constructions of each promoter. Figure S1. The structure of SLA and SLX. For SLA synthesis, N-acetylglucosamine-β1-3 galactosyltransferase (β1-3Gal-T 5) transfers a galactose (Gal) to an N-acetylglucosamine (GlcNac) with 1-3-linkage, resulting in the synthesis of type 1 chain, and then galactose-α2-3 sialyltransferase transfers a sialic acid (Sia) to the Gal residue of the type 1 with an 2-3 linkage. Finally, α1-4/3 fucosyltransferase transfers a fucose (Fuc) to the GlcNAc residue of the sialylated-type 1 with an 1-4 linkage to complete the synthesis of Siaα2-3Galβ1-3[Fucα1-4]GlcNAc structure. However, if N-acetylglucosamine-β1-4 galactosyltransferase I (β1-4Gal-T I) catalyzes the addition of UDP-galactose to terminal N-acetylglucosamine with 1-4-linkage, resulting in the synthesis of type 2 chain, continuously, galactose-α2-3 sialyltransferase transfers a sialic acid to the Gal residue of the type 2 chain with an 2-3 linkage. Finally, α1-4/3 fucosyltransferase transfers a fucose to the GlcNAc residue of the sialylated-type 2 chain with an 1-3 linkage to complete the synthesis of Siaα2-3Galβ1-4[Fucα1-3]GlcNAc structure. Figure S2. The enhanced expression and promoter activity of ST3Gal III, FUT III and VII genes in HBx-transfected cells. (A) Total RNA from each cell was isolated using the Trizol reagent. The mRNA expression of α2-3 sialyltransferases and α1-3/4 fucosyltransferases genes was detected by RT-PCR using primers indicated in Table 1. β-actin was included as an internal control. 1, Chang; 2, Chang pcDNA; 3, Chang pcDNA-HBx. (B) After transfection with each promoter, luciferase activity from the cells was analysed as described in Materials and Methods. Relative luciferase activity was normalized with the activity of pCMVβ-gal plasmid. (DOC 568 KB) [file 12943_2013_1425_MOESM1_ESM.doc]

**Additional files**

**Chung et al.**

**Table S1. The sequences of primers used in this study**

| Primers | Sequences | Strand |
| --- | --- | --- |
| HBx | 5'-ACGGAATTCATGGCTGCTAGGGTGT-3‘  5'-ACGGTCGACTTAGGCAGAGGTGAAA-3' | Sence  Antisence |
| ST3Gal I | 5’-CTGAAAGTGCTCACCTTCCTCGTGC-3’  5’-ACAGCTCCTTGATGGTGTCATTCAA-3’ | Sence  Antisence |
| ST3Gal II | 5’-TACCTGGACTCAGGGGCCCTGGATG-3’  5’-GGCACTGGTGGGGGTCCCGGAA -3’ | Sence  Antisence |
| ST3Gal III | 5’-ATGGGACTCTTGGTATTTGTGCGC-3’  5’-CATCCAGGAACATGGGTGCTGGCTT-3’ | Sence  Antisence |
| ST3Gal IV | 5’-CTTGAGGATTATTTCTGGGTCAAGA-3’  5’-TGTCAGCTGCAATCTCCATGAAGAA-3’ | Sence  Antisence |
| ST3Gal V | 5’-TGAAGCTGAGTCCAAGTACGATCCTCC-3’  5’-GTCGTACCCAGAATGGCAGGG-3’ | Sence  Antisence |
| ST3Gal VI | 5’-ATTCCTGAGTGCTGTCTTCCTCTAT-3’  5’-GTCAAACTCATCAAAGAGATCAC-3’ | Sence  Antisence |
| FUT III | 5’-ACTGGGATATCATGTCCAACCCTAAGTCAC-3’  5’-GGGCCAGGTCCTTGGGGCTCTGGAAGTCG-3’ | Sence  Antisence |
| FUT IV | 5’-GAGAGGCTCAGGCCGTGCTTTT-3’  5’-GCAGGAGCCCAATTTCGGGCAC-3’ | Sence  Antisence |
| FUT V | 5’-GAACCTGTCACCGGGGCTCCC-3’  5’- GGGCCAGGTCCTTGGGGCTCTGGAAGTCG-3’ | Sence  Antisence |
| FUT VI | 5’-AATGGGTCCCGCTTCCCAGACAG-3’  5’-GGGCCAGGTCCTTGGGGCTCTGGAAGTCG-3’ | Sence  Antisence |
| FUT VII | 5’-ATCTTCAACTGGGTGCTGAG-3’  5’-GTTGGTATCGGCTCTCATTC-3’ | Sence  Antisence |
| β1-3Gal-T 5 | 5’-AACGAAAGAGGTGGACCAGG-3’  5’-CCCACAAACACGTCTTCCAG-3’ | Sence  Antisence |
| β1-4Gal-T I | 5’-GTGACGTGGACCTCATTCCA-3’  5’-TCTTGTCTCTTGAGTGGCGG-3’ | Sence  Antisence |

**Table S2. The sequences of primers for constructions of each promoter**

| Primers | Sequences | Restriction enzyme | Strand |
| --- | --- | --- | --- |
| ST3Gal III |  |  |  |
| 870 | 5’-GGTACCCGACCTCAAGAGAACTGGCCCCAGC-3’ | Kpn I | Sense |
| 543 | 5’-GGTACCTTCTACGAGTCTCTTAACGCTCACC-3’ | Kpn I | Sense |
| 306 | 5’-GGTACCGGAAGAGGGTTCCTCTAGACAGCTC-3’ | Kpn I | Sense |
|  | 5’-AGATCTCCACAACGCGCTGGGCTGCATGGGC-3’ | Bgl II | Antisense |
| FUT III |  |  |  |
| 741 | 5’-ATGAGCTCTCTCAAGGGTGACGAGGGTATATTG-3’ | Sac1 | Sense |
| 591 | 5’-ATGAGCTCCATCAGTTAAGGCAGGAACTGGC-3’ | Sac1 | Sense |
| 391 | 5’-ATGAGCTCATGCAATAGGTGCATAAGCAATGTTTG-3’ | Sac1 | Sense |
| 241 | 5’-ATGAGCTCCGTTTTCACTTTATGGATGGACCAG-3’ | Sac1 | Sense |
| 91 | 5’-ATGAGCTCCCAGACAATATCCCTGCTGCAG-3’ | Sac1 | Sense |
|  | 5’-ATCTCGAGCTGGAGAGGCAGCGAGCAGT-3’ | Xho1 | Antisense |
| FUT VII |  |  |  |
| 850 | 5’-ATGGTACCGGGGAGTCTGGACTTCAGGC-3’ | KpnI | Sense |
| 540 | 5’-ATGGTACCGGGTTCCTAGATGGCAGAGGTGG-3’ | KpnI | Sense |
| 350 | 5’-ATGGTACCAAGTGCACTCGCTGTCTGCAG-3’ | KpnI | Sense |
| 193 | 5’-ATGGTACCCTCTGGAGACCAGTGGGGTG-3’ | KpnI | Sense |
|  | 5’-ATCTCGAGTGCCCAAGGTGAGGGAACTG-3’ | XhoI | Antisense |
| β-1,3 GalT 5 |  |  |  |
| 663 | 5’-ATGGTACCCCAGTCCTTAGAGAGGTGGACTTC-3’ | KpnI | Sense |
| 303 | 5’-ATGGTACCGAGGAGCCTGCAGCAGGCAG-3’ | KpnI | Sense |
|  | 5’-ATCTCGAGTGGGCCCAGTCAATGGATGG-3’ | XhoI | Antisense |


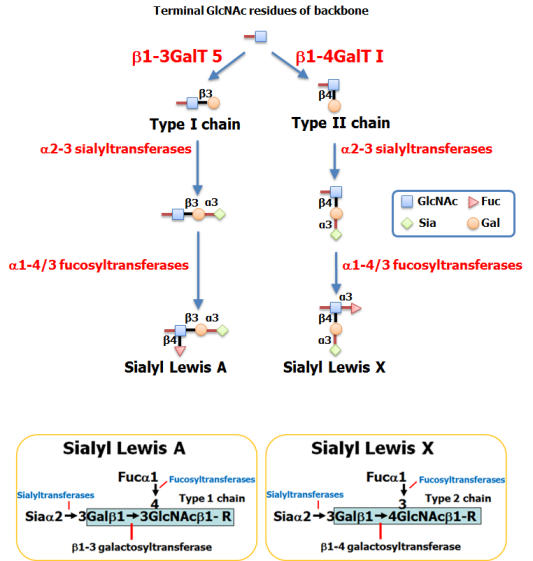


**Figure S1. The structure of SLA and SLX.** For SLA synthesis, *N*-acetylglucosamine-β1-3 galactosyltransferase (β1-3Gal-T 5) transfers a galactose (Gal) to an N-acetylglucosamine (GlcNac) with 1-3-linkage, resulting in the synthesis of type 1 chain, and then galactose-α2-3 sialyltransferase transfers a sialic acid (Sia) to the Gal residue of the type 1 with an 2-3 linkage. Finally, α1-4/3 fucosyltransferase transfers a fucose (Fuc) to the GlcNAc residue of the sialylated-type 1 with an 1-4 linkage to complete the synthesis of Siaα2-3Galβ1-3[Fucα1-4]GlcNAc structure. However, if N-acetylglucosamine-β1-4 galactosyltransferase I (β1-4Gal-T I) catalyzes the addition of UDP-galactose to terminal N-acetylglucosamine with 1-4-linkage, resulting in the synthesis of type 2 chain, continuously, galactose-α2-3 sialyltransferase transfers a sialic acid to the Gal residue of the type 2 chain with an 2-3 linkage. Finally, α1-4/3 fucosyltransferase transfers a fucose to the GlcNAc residue of the sialylated-type 2 chain with an 1-3 linkage to complete the synthesis of Siaα2-3Galβ1-4[Fucα1-3]GlcNAc structure.

**
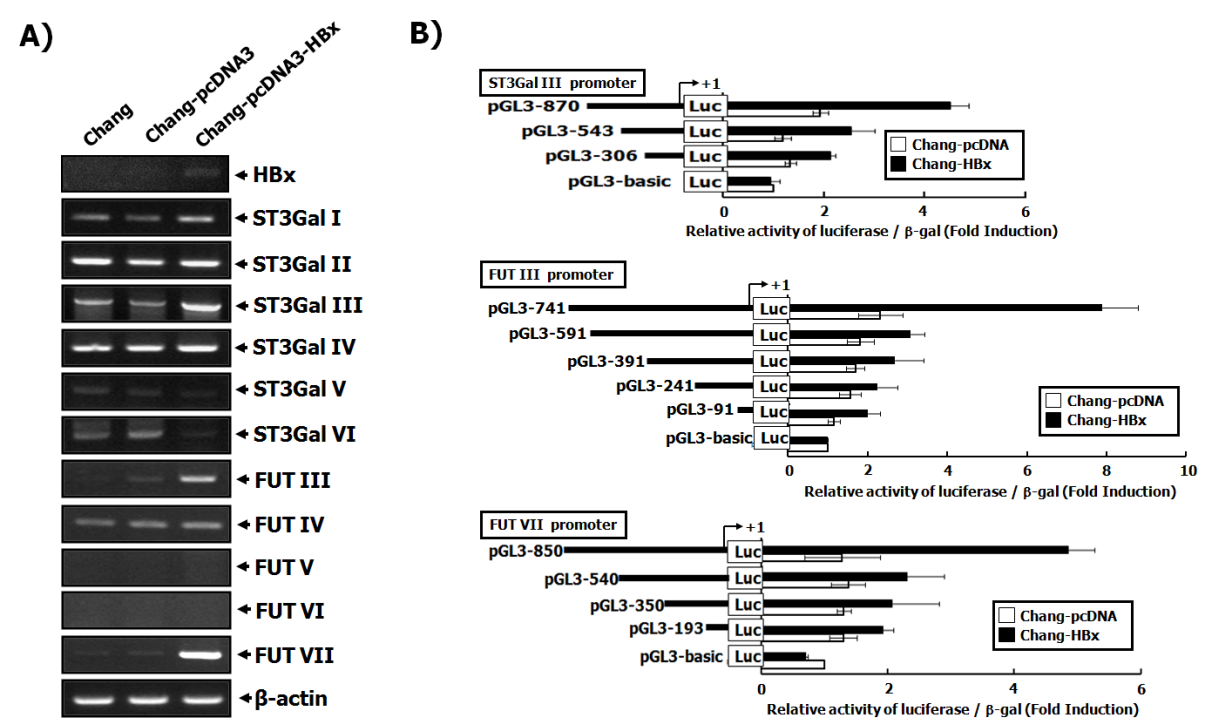
**

**Figure S2. The enhanced expression and promoter activity of ST3Gal III, FUT III and VII genes in HBx-transfected cells.** (A) Total RNA from each cell was isolated using the Trizol reagent. The mRNA expression of α2-3 sialyltransferases and α1-3/4 fucosyltransferases genes was detected by RT-PCR using primers indicated in Table I. β-actin was included as an internal control. 1, Chang; 2, Chang pcDNA; 3, Chang pcDNA-HBx. (B) After transfection with each promoter, luciferase activity from the cells was analysed as described in Materials and Methods. Relative luciferase activity was normalized with the activity of pCMVβ-gal plasmid.


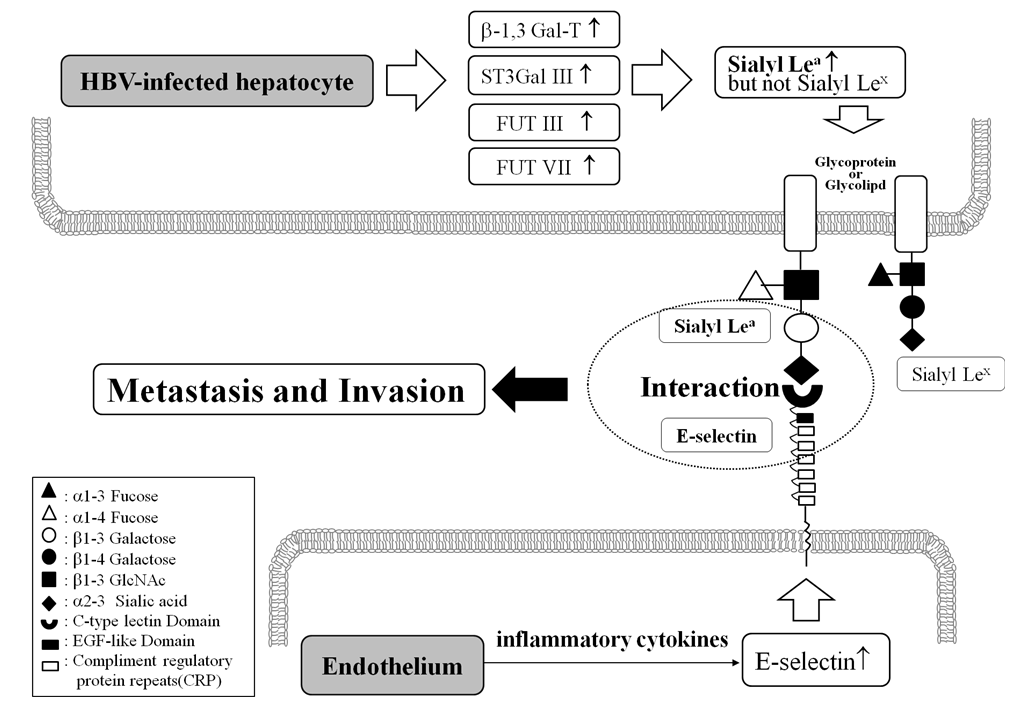


**Fig. S3. Schematic diagram illustrating the adhesion of HBx-transfected cells to endothelial cells, resulting from the interaction of SLA antigen synthesized on the surface of HBx-transfected cells with E-selectin expressed on the surface of TNF-α-stimulated endothelial cells.**
